# Quantitative, noninvasive MRI characterization of disease progression in a mouse model of non-alcoholic steatohepatitis

**DOI:** 10.1038/s41598-021-85679-4

**Published:** 2021-03-17

**Authors:** Philip A. Waghorn, Diego S. Ferreira, Derek J. Erstad, Nicholas J. Rotile, Ricard Masia, Chloe M. Jones, Chuantao Tu, Mozhdeh Sojoodi, Yin-ching I. Chen, Franklin Schlerman, Jeremy Wellen, Robert V. P. Martinez, Kenneth K. Tanabe, Bryan C. Fuchs, Peter Caravan

**Affiliations:** 1 Department of Radiology, Massachusetts General Hospital, Athinoula A. Martinos Center for Biomedical Imaging, Institute for Innovation in Imaging, Harvard Medical School, 149 13th St., Boston, MA 02129 USA; 2 Division of Surgical Oncology, Massachusetts General Hospital Cancer Center, Harvard Medical School, Boston, MA 02114 USA; 3 Pfizer, Cambridge, MA 02139 USA; 4 Present Address: School of Pharmacy, Universidade Federal de Minas Gerais, Av. Presidente Antônio Carlos, 6627, Pampulha, Belo Horizonte, Minas Gerais Brazil

**Keywords:** Hepatology, Liver, Liver diseases, Magnetic resonance imaging

## Abstract

Non-alcoholic steatohepatitis (NASH) is an increasing cause of chronic liver disease characterized by steatosis, inflammation, and fibrosis which can lead to cirrhosis, hepatocellular carcinoma, and mortality. Quantitative, noninvasive methods for characterizing the pathophysiology of NASH at both the preclinical and clinical level are sorely needed. We report here a multiparametric magnetic resonance imaging (MRI) protocol with the fibrogenesis probe Gd-Hyd to characterize fibrotic disease activity and steatosis in a common mouse model of NASH. Mice were fed a choline-deficient, L-amino acid-defined, high-fat diet (CDAHFD) to induce NASH with advanced fibrosis. Mice fed normal chow and CDAHFD underwent MRI after 2, 6, 10 and 14 weeks to measure liver T1, T2*, fat fraction, and dynamic T1-weighted Gd-Hyd enhanced imaging of the liver. Steatosis, inflammation, and fibrosis were then quantified by histology. NASH and fibrosis developed quickly in CDAHFD fed mice with strong correlation between morphometric steatosis quantification and liver fat estimated by MRI (r = 0.90). Sirius red histology and collagen quantification confirmed increasing fibrosis over time (r = 0.82). Though baseline T1 and T2* measurements did not correlate with fibrosis, Gd-Hyd signal enhancement provided a measure of the extent of active fibrotic disease progression and correlated strongly with lysyl oxidase expression. Gd-Hyd MRI accurately detects fibrogenesis in a mouse model of NASH with advanced fibrosis and can be combined with other MR measures, like fat imaging, to more accurately assess disease burden.

## Introduction

Nonalcoholic fatty liver disease (NAFLD) is fast becoming one of the most prominent causes of liver disease^1^ with expectations that it will soon be the leading indication of liver transplantation^2^. Between 20–30% of adults in the western world are now estimated to have NAFLD^3^, and between 5–6% of those patients with NAFLD will develop non-alcoholic steatohepatitis (NASH)^4^ in which substantial liver injury and inflammation are present^5^. While patients with NAFLD have good long-term prognosis, with no increased liver related morbidity or mortality^6^, those with NASH have increased risk of cirrhosis, hepatocellular carcinoma and liver or cardiovascular related mortality^7^. The financial burden of NAFLD and NASH healthcare management is currently estimated to cost in excess of $100 billion in the USA alone^8^. There is therefore a need to identify those NAFLD patients who are at risk of developing NASH and cirrhosis so as to better manage patient healthcare through improved lifestyle, exercise and diet^9,10^. In addition, a large number of new therapies have entered clinical trials^11,12^, however there have been a number of failures pointing to the challenge of designing safe, effective therapies for this complex disease^13^.

There remains an unmet need for improved diagnostics to better stratify patients into clinical trials and to accurately monitor response to therapy^14,15^. Preclinically, there is also a need for better tools to allow longitudinal, noninvasive, quantitative characterization of NASH disease models, where historically treatment response has been assessed ex vivo. Disease progression in animal models is heterogeneous and thus at the time of treatment initiation some animals may have severe disease while others have mild disease, making it challenging to assess treatment effects. Multiparametric imaging can potentially provide a three dimensional assessment of the entire liver and can be performed prior to, and at multiple timepoints following treatment to assess the time course and the effect of the therapeutic intervention.

Liver biopsy is the gold standard for NASH diagnosis^16,17^ and the only current way to distinguish hepatic steatosis (intracellular fat content in > 5% hepatocytes) from steatohepatitis (distinct morphological features including hepatocyte ballooning, leukocytes and perisinusoidal fibrosis)^18^. Biopsy however is an imperfect method with appreciable sampling error, high inter-observer variability, and risk of complications^19,20^. Transient elastography (TE)^21^ and magnetic resonance elastography (MRE)^22^ both reliably detect moderate and advanced liver fibrosis, but these methods cannot reliably distinguish simple steatosis from NASH^23^. Combining three-dimensional MRE (3D-MRE) with magnetic resonance imaging proton density fat fraction (MRI-PDFF) has shown more promise predicting NASH disease activity by the non-alcoholic fatty liver disease activity score (NAS)^24^. Recently, a multiparametric MRI algorithm, called LiverMultiScan, that assesses liver fat, T2, and iron-corrected T1 was shown to be superior to liver stiffness for stratifying patients with simple steatosis from those with NASH, but LiverMultiScan could not accurately stage fibrosis^25^.

For the last several years, our group has been developing molecular MRI to quantify the collagen deposition that occurs during scar tissue formation^26–35^. More recently, we have developed the gadolinium-based probe Gd-Hyd to allow for MR imaging of the allysine concentration in tissue which is responsible for the cross-linking and stabilization of collagen proteins during scar tissue formation^36–38^.

The choline-deficient, L-amino acid defined, high fat diet (CDAHFD) mouse model is increasingly used as a preclinical model to test novel NASH therapies. The model is characterized by hepatic steatosis that gives way to increasing fibrosis with duration on diet, as well as a persistent inflammatory component. The goal of this study was to combine Gd-Hyd imaging of fibrogenesis with other MR measurements to create a multiparametric MRI exam to non-invasively assess the natural history of disease progression and resolution in the mouse CDAHFD model. The ultimate aim of this work is to develop a comprehensive MRI protocol to quantify steatosis and fibrogenesis in NASH patients without the need for biopsy.

## Methods

### Probe

**Figure 1:**
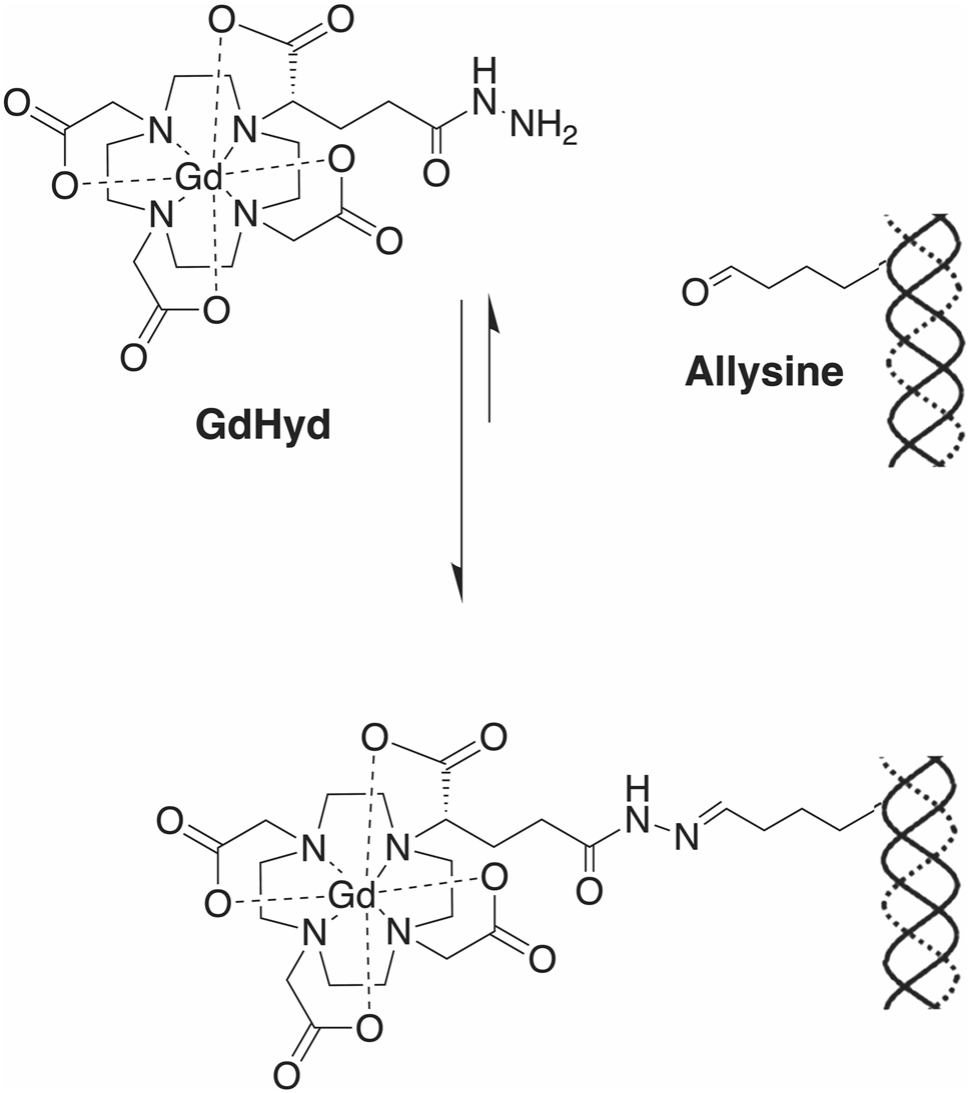
Structure of Gd-Hyd. Gd-Hyd is a water soluble, low molecular weight, extracellular gadolinium-based imaging agent functionalized with a hydrazide moiety for binding allysine on collagen.

Gd-Hyd (Fig. 1) is a water soluble, low molecular weight, extracellular gadolinium-based probe that targets allysine which is generated by oxidation of lysine residues on matrix proteins by lysyl oxidase enzymes^36^. Gd-Hyd was shown to bind to allysine containing proteins and to allysine rich porcine aorta (K_d_ = 650 μM), and was used to detect lung fibrogenesis in a bleomycin induced mouse model^36^ and liver fibrogenesis in CCl_4_ and CDAFHD models^36–38^. The gadolinium core provides MR signal enhancement on allysine binding (relaxivity = 16.2 mM^−1^ s^−1^ at 1.4 T when bound to protein vs 4.1 mM^−1^ s^-1^ when unbound).

### Animal model

All experiments were performed in accordance with the NIH Guide for the Care and Use of Laboratory Animals and in compliance with the ARRIVE guidelines^39^, and approved by the MGH Institutional Animal Care and Use Committee. A total of 70 mice were used in this study and randomized to each study group. To induce NASH, 6-week old, male C57BL/6 mice (Charles River Labs, Wilmington, MA) were fed a CDAHFD consisting of 60 kcal% fat and 0.1% methionine by weight as previously described^40^. Three groups of mice were studied: Group 1 mice were fed normal chow for 2 (n = 6), 6 (n = 6), 10 (n = 9) or 14 weeks (n = 5); Group 2 mice were fed CDAHFD for 2 (n = 6), 6 (n = 6), 10 (n = 12) or 14 weeks (n = 12); and Group 3 mice were fed CDAHFD for 10 weeks followed by normal chow for 4 weeks (n = 8). No animals were excluded from the study.

### MR imaging and analysis

Animals were anesthetized with isoflurane (1–2%) and placed in a specially designed cradle with body temperature maintained at 37 °C. Anesthesia was adjusted to maintain a respiration rate of 60 ± 5 breaths per minute. The tail vein was cannulated for intravenous (i.v.) delivery of the contrast agent while the animal was positioned in the scanner. Imaging was performed at 9.4 T using a small bore animal scanner with a custom-built volume coil. Mice were imaged with a dose of 200 μmol/kg of Gd-Hyd.

A series of baseline images (T1- and T2*-mapping sequences^41^, two-point Dixon sequence^42^, and 2D T1 weighted dynamic contrast enhanced (DCE) imaging) were first acquired, then a bolus (maximum volume 100 μL) of Gd-Hyd was administered i.v. and imaging performed for a period of 30 min post injection. Following the imaging session, animals were euthanized (45 min post injection), and liver and other tissues were removed for further analysis.

T1-mapping sequence: Rapid Acquisition with Relaxation Enhancement (RARE) inversion recovery (IR), TR/TE = 5000/7.27 ms, matrix = 195 × 195, FOV = 2.5 × 2.5 cm, 9 inversion times of 0, 300, 550, 700, 850, 1500, 3000, 5000, and 7000 ms, single slice (1 mm), RARE factor 16. T1 was quantified from a three parameter fit of the dependence of liver signal intensity (SI) on inversion time (TI). T2*-mapping sequence: Multi-echo gradient-echo, TR = 1500 ms, matrix = 128 × 128, flip angle = 90°, FOV = 2.56 × 2.56 cm, 2 averages, 12 echo times of 2.62, 5.17, 7.72, 10.27, 12.82, 15.38, 17.93, 20.48, 23.03, 25.58, 28.13, 30.68 ms. T2* maps were generated from exponential fitting of the signal intensity as a function of the gradient-echo time (TE). Two-point Dixon sequence: Conventional gradient echo sequence with TR = 500 ms, TE (in-phase) = 1.06 ms and TE (out-of-phase) = 1.41 ms, FOV = 2.4 × 2.4 cm, matrix = 96 × 96, 18 × 1 mm slices, flip angle = 30°. 2D T1 weighted dynamic contrast enhanced (DCE) images were acquired prior to and at 5, 15 and 25 min following intravenous administration of probes. TR/TE = 41/2.5 ms, temporal resolution = 2.64 ms, number of repetitions = 54, matrix = 64 × 64, flip angle = 60° and FOV = 4.385 × 2.644 cm. For DCE analysis region of interests (ROIs) were segmented using FreeView (FreeSurfer software, v7.1.0, General Hospital Corporation, Boston, MA, https://surfer.nmr.mgh.harvard.edu/fswiki/FreeviewGuide). Blood vessels were excluded from the liver ROI via the signal threshold method. The mean and standard deviation of the MR signal over an ROI was then calculated. The signal to noise ratio (SNR) for liver and muscle ROIs were established using SNR_liver_ = mean(SI_liver_)/stdev_noise_, and SNR_muscle_ = mean(SI_muscle_)/stdev_noise_.

### Gene expression

Quantification of lysyl oxidase gene expression was performed as previously described^43^.

### Tissue analysis

Formalin-fixed samples were embedded in paraffin, cut into 5 µm-thick sections and stained with hematoxylin–eosin (H-E) and Sirius red according to standard procedures. The grading of steatosis, ballooning^44^ and fibrosis^45^ was performed in a blinded manner by a board-certified pathologist with dedicated expertise in liver pathology. For NASH components, H-E sections were evaluated. The grade of steatosis was quantified as grade 0: < 5%, grade 1: 5–33%, grade 2: 33–66%, and grade 3: > 66%, the grade of inflammation was quantified as grade 0: no foci, grade 1: < 2 foci/20X field, grade 2: 2–4 foci/20X field, and grade 3: > 4 foci/20X field, and the grade of ballooning was quantified as grade 0: no ballooning, grade 1: few ballooned cells, and grade 2: many cells/prominent ballooning. For fibrosis, Sirius red stained sections were evaluated and the grade of fibrosis quantified as grade 0: none, grade 1A: mild pericellular, zone 3, grade 1b: moderate pericellular, zone 3, grade 1c: any pericellular, zone 1, grade 2: pericellular zone 1 and 3, grade 3: bridging, and grade 4: cirrhosis. In addition, collagen proportional area (CPA) was morphometrically quantified on whole slide scanned Sirius red sections with image processing software (ImageJ, NIH) according to our established protocol^30,34,41,46^. Hepatic lipid vacuolization (LV) was calculated from whole slide scanned H-E sections using ImageJ.

Hydroxyproline in tissue was quantified by HPLC analysis using a reported method^47^, and was expressed as amounts per wet weight of tissue.

### Statistics

All data are shown as mean ± SEM. Differences between two groups were tested with unpaired Student’s t-Test, and differences among more than two groups were tested with one-way analysis of variance (ANOVA) followed by Tukey’s post-hoc test with *p* < 0.05 considered as significant.

## Results

### NASH and fibrosis develop quickly in CDAHFD fed mice

The main objective of this study was to image fibrogenesis as fibrosis stage is the only histological feature of disease that is associated with worse outcomes in NASH^48^. However, fibrosis development is weak in traditional animal models of obesity and fatty liver disease, like high fat or western diets^49^. We therefore chose to use a CDAHFD for these studies as NASH and fibrosis develop quickly in mice fed CDAHFD.

**Figure 2:**
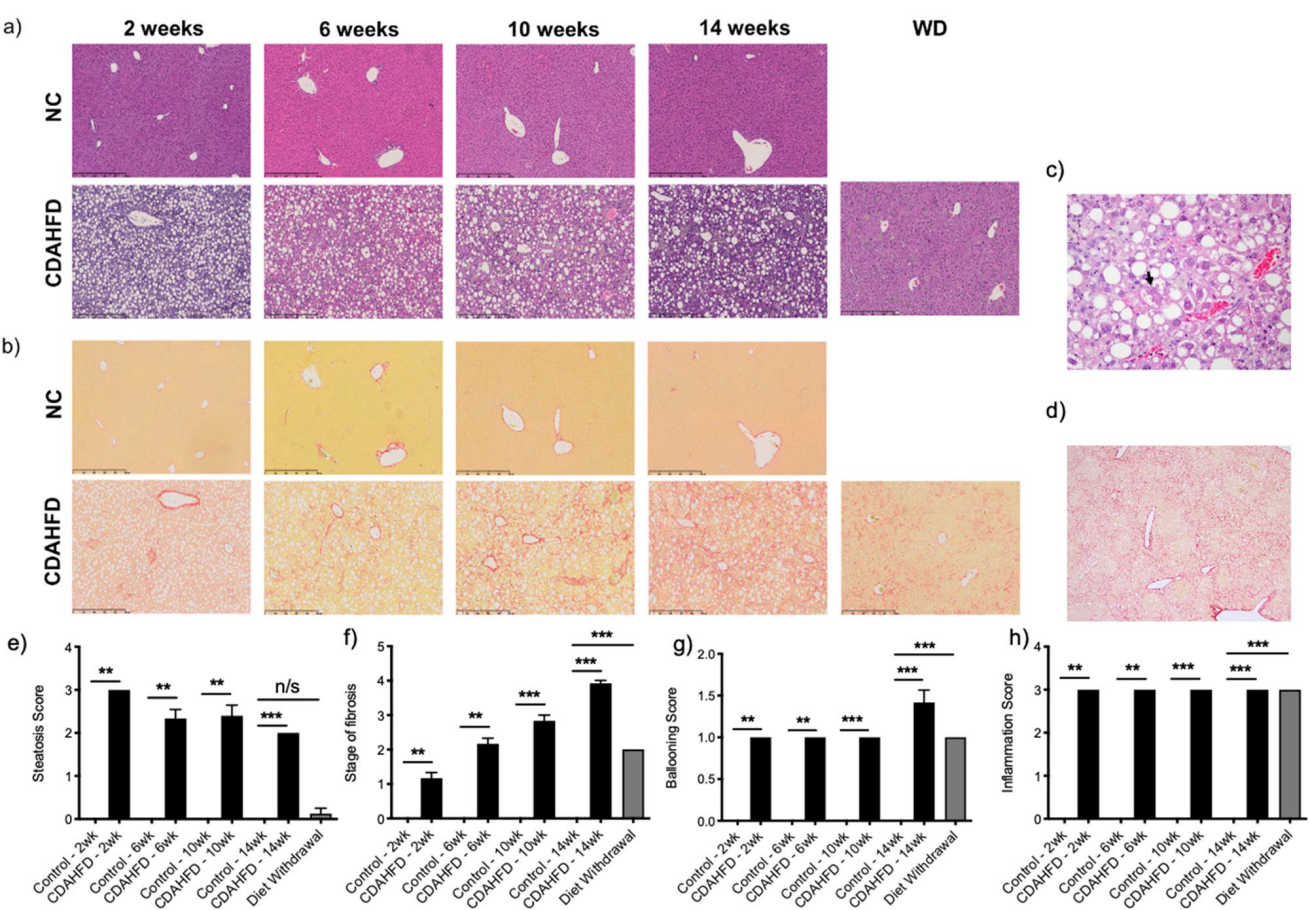
Disease progression in the mouse CDAHFD model. Representative (**a**) H&E and (**b**) Sirus red staining of livers from normal chow (NC) control and CDAHFD fed mice after 2, 6, 10 and 14 weeks of diet and after withdrawal of diet (WD) between week 10 and week 14. Higher magnification (**c**) H&E and (**d**) Sirius red pictures are shown to illustrate ballooning and cirrhosis, respectively. Arrow in (**c**) indicates Mallory’s hyaline. Comparison of (**e**) steatosis score, (**f**) stage of fibrosis, (**g**) ballooning score and (**h**) inflammation score in control and CDAHFD fed mice after 2, 6, 10 and 14 weeks and after WD. (*P < 0.05, **P < 0.01, ***P < 0.001, *n/s* not significant).

Male C57BL/6 mice were fed control or CDAHFD diet and subsets of animals were imaged and sacrificed after 2, 6, 10, and 14 weeks. Steatosis developed quickly (Fig. 2a) with the CDAHFD diet and all animals were scored as Grade 3 after 2 weeks (Fig. 2e). As the time on the CDAHFD diet progressed, the liver became more fibrotic while steatosis actually decreased with animals being scored as either Grade 2 or 3 at 6 and 10 weeks, and exclusively Grade 2 by 14 weeks (Fig. 2e). Inflammation also progressed rapidly on CDAHFD with all animals scored Grade 3 at week 2. Interestingly, the inflammation persisted throughout the model with all animals scored as Grade 3 (Fig. 2h). A few ballooned cells (Grade 1) characterized by cytoplasmic degeneration without fat droplets within the cytoplasm could be seen in all animals at every time point with some animals at the 14 weeks having prominent ballooning (Grade 2) characterized by enlarged hepatocytes with cytoplasmic degeneration and accumulation of Mallory’s hyaline (Fig. 2c,g).

Fibrosis development in the CDAHFD model was reproducible between animals and rapidly progressed (Fig. 2b,f). After 2 weeks on CDAHFD, all animals had mild Zone 3 pericellular fibrosis (Grade 1a) except for one animal who had pericellular fibrosis in Zones 1 and 3 (Grade 2). After 6 weeks on CDAHFD, all animals had pericellular fibrosis in Zones 1 and 3 (Grade 2) except for one animal who had progressed to portal-central bridging fibrosis (Grade 3). After 10 weeks on CDAHFD, all animals had bridging fibrosis (Grade 3) except for one animal that had moderate pericellular fibrosis in Zone 3 (Grade 1b). After 14 weeks on CDAHFD, all animals had cirrhosis (Grade 4) characterized by diffuse bridging fibrosis and nodule formation. Interestingly, the fibrosis at all time points consistently had a “chickenwire” meshwork-like quality (delicate pericellular and sinusoidal fibrosis) characteristic of early NASH, without forming the denser, thicker, more defined portal-central bridges often seen in advanced NASH in humans. However, the “chickenwire” meshwork-like fibrosis became somewhat denser and more confluent over time, connecting the expanded fibrotic portal tracts and central veins, and expansile nodules were observed by 14 weeks (Fig. 2d).

In a separate experiment, animals were fed CDAHFD for 10 weeks and then switched to control diet and imaged and sacrificed at 14 weeks as a model of disease resolution. Steatosis completely resolved in these animals except for one animal that scored Grade 1 (Fig. 2e). Likewise, while some animals fed CDAHFD for 14 weeks had Grade 2 ballooning, all animals switched to control diet after 10 weeks had Grade 1 ballooning (Fig. 2g). Interestingly, inflammation was still present in these animals but the types of inflammatory cells present were almost exclusively lipid-filled histiocytes (Fig. 2h). By analogy to human liver biopsies, where previous hepatocyte injury may be detected as histiocytes containing debris from engulfed dead hepatocytes (so-called ceroid-laden macrophages), the lipid-filled histiocytes in these animals could perhaps be the sequelae of prior hepatocyte injury (i.e. containing engulfed dead steatotic hepatocytes) rather than an indication of active inflammation. Finally, as expected, the fibrosis regressed to pericellular fibrosis in Zones 1 and 3 (Grade 2) for every animal (Fig. 2f).

### Strong correlation between morphometric steatosis quantification and MR fat imaging

**Figure 3:**
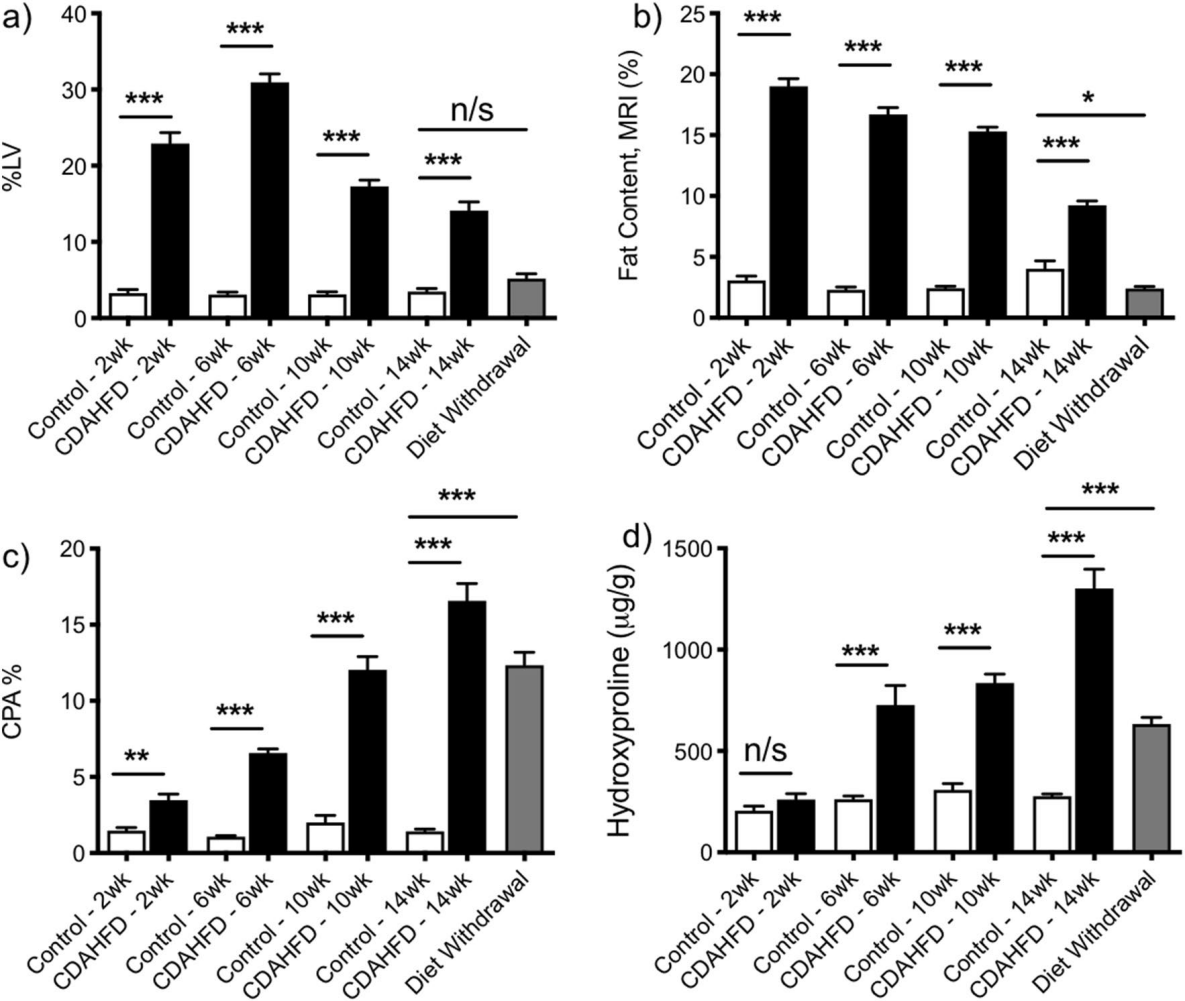
Non-contrast MRI Measurements of fibrosis and steatosis. Comparison of (**a**) lipid vacuolization (LV), (**b**) MRI fat content (%), (**c**) % collagen proportional area (CPA) and (**d**) hydroxyproline content in control and CDAHFD groups after 2, 6, 10 and 14 weeks and after CDAHFD withdrawal between week 10 and week 14. (*P < 0.05, **P < 0.01, ***P < 0.001, *n/s* not significant).

Morphometric quantification of steatosis on H&E slides shows that for animals on CDAHFD there is an immediate increase in the LV after only 2 weeks compared to animals fed on normal chow (LV = 22.90 ± 1.45% vs 3.28 ± 0.48%, p < 0.0001). LV continued to increase in the CDAHFD groups up to 6 weeks (30.91 ± 1.11%) followed by a steady decrease at the 10 (17.28 ± 0.82%) and 14 (14.10 ± 1.35%) week time-points. By comparison, LV for mice on a normal diet remained consistently below 4% for the whole 14 weeks (Fig. 3a). Consistent with the morphometric quantification of steatosis, MRI revealed a significantly increased fat percentage (%fat) for the 2 week animals in the CDAHFD group (%fat = 19.00 ± 0.64 vs 2.41 ± 0.17, p < 0.0001), with the fat percentage decreasing over the course of 14 weeks (Fig. 3b). Compared to those animals on CDAHFD for the entire 14 weeks, switching the diet back to normal chow from week 10 to week 14 led to a significant decrease in both LV (14.10 ± 1.35% vs 5.17 ± 0.66%, p < 0.0001) and %fat (9.24 ± 0.35 vs 2.41 ± 0.17, p < 0.0001). There was no or little significant difference in LV or %fat, respectively, between the withdrawal group mice and those animals on normal diet for 14 weeks (LV: 5.17 ± 0.66 vs. 3.47 ± 0.42, p = 0.0875 and %fat: 2.41 ± 0.17 vs 4.04 ± 0.64, p = 0.011).

### Non-contrast MRI measurements do not correlate with fibrosis development

Sirius red staining of collagen confirmed that fibrosis increased over time for mice in the CDAHFD group with maximum collagen deposition at 14 weeks (Fig. 3c). Significant collagen deposition, as assessed morphometrically by collagen proportional area (CPA), was observed for CDAHFD mice compared to controls at 2 (3.48 ± 0.40 vs.1.47 ± 0.21, p = 0.0012), 6 (6.57 ± 0.27 vs. 1.08 ± 0.07, p < 0.0001), 10 (12.04 ± 0.91 vs. 2.02 ± 0.46, p < 0.0001) and 14 (16.57 ± 1.19 vs. 1.43 ± 0.16, p < 0.0001) weeks.

Quantitative analysis of hydroxyproline (Hyp) was used as an additional measure of the total amount of collagen in tissue (Fig. 3d). Hydroxyproline levels showed no significant response to CDAHFD at week 2 (control: 206 ± 23 μg/g, CDAHFD: 260 ± 29 μg/g, *p* = 0.1702), but increased steadily over time compared to control animals, which remained relatively constant, and were greatest at week 14 (control: 276.4 ± 11.2 μg/g, CDAHFD: 1300 ± 95 μg/g, *p* < 0.0001). Switching mice that had been fed CDAHFD for 10 weeks to normal chow for 4 weeks caused a significant decrease in collagen levels compared to those animals that received CDAHFD for the entire 14 weeks. CPA decreased to 12.34 ± 0.85% and hydroxyproline levels dropped to 634 ± 32 μg/g by week 14. However, contrary to steatosis, collagen levels did not reduce down to the levels seen for those animals on a normal diet for 14 weeks.

**Figure 4:**
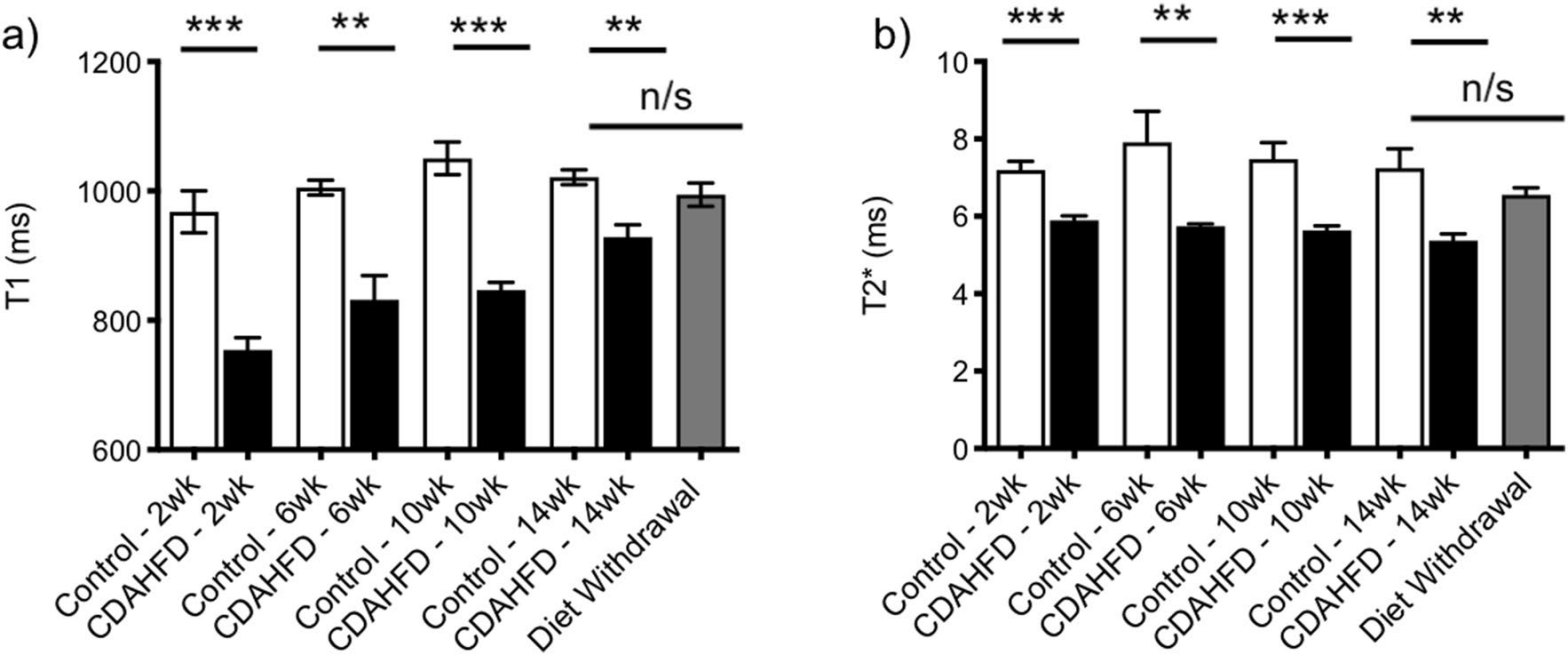
Relaxation time measurements (before Gd-Hyd injection) in the mouse CDAHFD model. Comparison of (**a**) T1 and (**b**) T2* values in control and CDAHFD groups after 2, 6, 10 and 14 weeks and after switching mice fed CDAHFD to normal chow between week 10 and week 14 (Diet Withdrawal). (*P < 0.05, **P < 0.01, ***P < 0.001, *n/s* not significant).

For mice on CDAHFD, the baseline liver T1 value (measured before Gd-Hyd injection) at 2 weeks was significantly reduced compared to the liver T1 of mice on normal diet (control: 968 ± 32 ms, CDAHFD: 754 ± 19 ms, *p* = 0.0002). Prolonged exposure to CDAHFD led to an increase in T1 value over time, with measurements at 14 weeks reaching similar levels to those of mice on the normal diet (control: 1020 ± 11 ms, CDAHFD: 925 ± 24 ms, *p* = 0.012). Switching mice fed a CDAHFD for 10 weeks to normal chow for 4 weeks increased the liver T1 value even further compared to those mice fed CDAHFD for the entire 14 weeks, with no significant difference between mice on diet withdrawal and mice on normal chow (control: 1020 ± 11 ms, withdrawal: 944 ± 18 ms, *p* = 0.46). Baseline T1 values did not provide a reliable method to distinguish the extent of fibrosis in the CDAHFD model (Fig. 4a), and if anything, more closely tracked with steatosis.

Liver T2* values were significantly decreased for mice fed CDAHFD compared to mice fed normal diet at all time points, but showed only minimal differences over the course of the diet (CDAHFD: 5.90 ± 0.11 ms at 2 weeks vs 5.37 ± 0.18 ms at 14 weeks, control: 7.20 ± 0.23 ms at 2 weeks vs 7.24 ± 0.50 ms at 14 weeks). Switching mice fed a CDAHFD for 10 weeks to normal chow for 4 weeks led to a small but significant increase in liver T2* signal compared to mice fed CDAHFD for the entire 14 weeks (p = 0.0005), with little difference between mice on diet withdrawal and mice on normal chow (control: 7.24 ± 0.50 ms, withdrawal: 6.56 ± 0.18, p = 0.048). Baseline T2* measurements also did not provide a reliable method to distinguish the extent of fibrosis in the CDAHFD model (Fig. 4b).

### Gd-Hyd detects fibrosis in CDAHFD mice

Based on the results from our previous CCl_4_ study^36^, we reasoned that immediately after Gd-Hyd injection, the liver in all animals would enhance because of a blood pool effect, but as the blood Gd-Hyd concentration decreased (t_1/2_ = 5 min) differences between the fibrotic CDAHFD and control livers for the allysine-targeted agent Gd-Hyd would become visible. Since the kinetics of NASH liver washout with Gd-Hyd was unknown, T1 weighted DCE measurements were performed prior to, and at multiple time points out to 30 min post injection.

**Figure 5:**
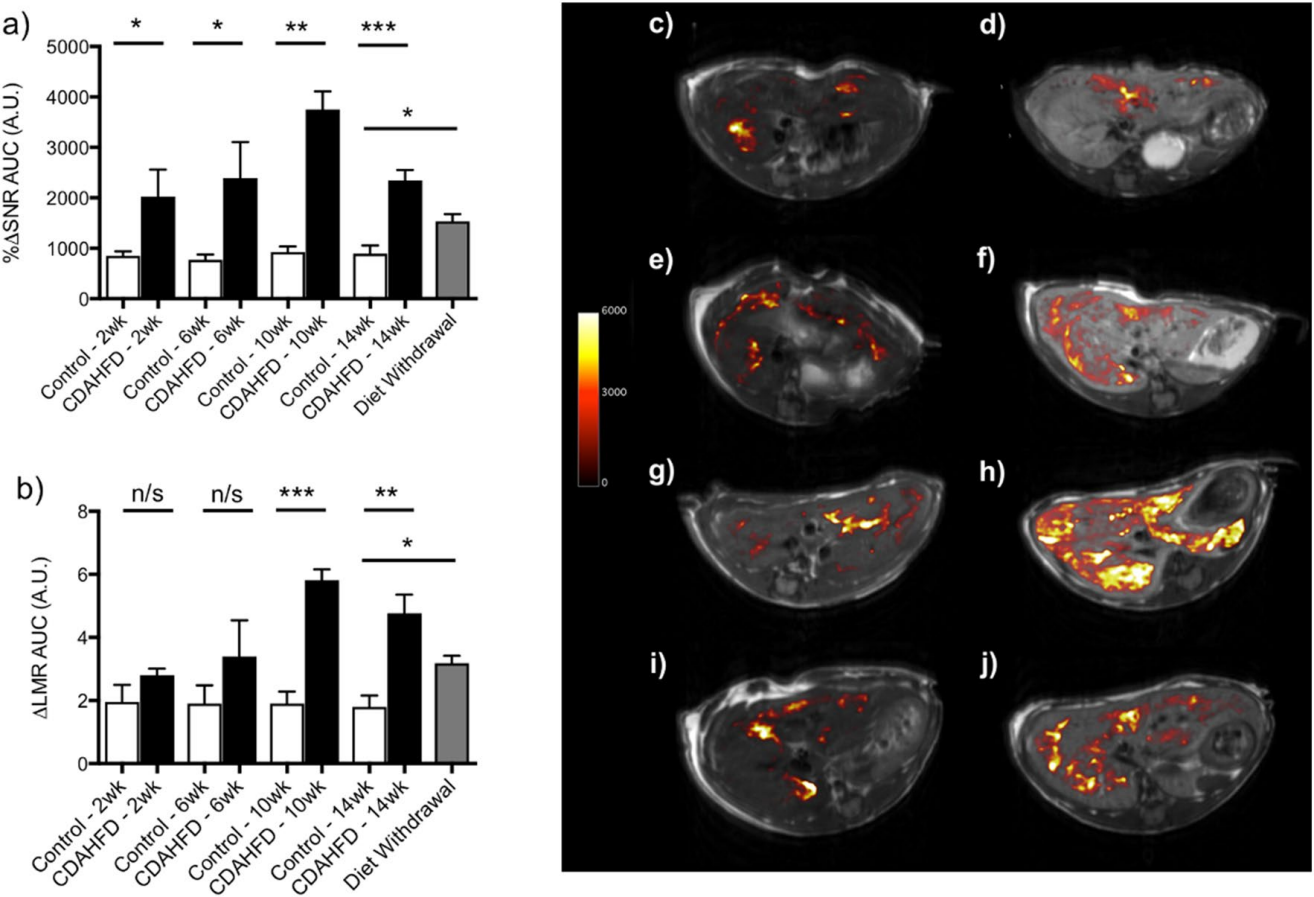
Molecular MR imaging of allysine in the CDAHFD model. Comparison of the post injection Gd-Hyd signal increase for control and CDAHFD groups after 2, 6, 10 and 14 weeks and switching mice fed CDAHFD to normal chow between week 10 and week 14 (Diet Withdrawal) represented as (**a**) the AUC of the percentage increase in signal, %ΔSNR vs time and (**b**) AUC of the liver signal to muscle signal ratio, ΔLMR vs time. (*P < 0.05, **P < 0.01, ***P < 0.001, *n/s* not significant). Axial MR images with overlaid liver enhancement (shown as the 25 min post-injection–pre-injection difference image in false color) for Gd-Hyd in mice after being fed (**c**) normal chow for 2 weeks, (**d**) CDAHFD for 2 weeks, (**e**) normal chow for 6 weeks, (**f**) CDAHFD for 6 weeks, (**g**) normal chow for 10 weeks, (**h**) CDAHFD for 10 weeks, (**i**) CDAHFD for 10 weeks with switching to normal diet for 4 weeks (Diet Withdrawal) and (**j**) CDAHFD for 14 weeks.

To quantify the amount of probe in vivo the signal in the liver and adjacent skeletal muscle were measured. The signal to noise ratio (SNR) in liver pre- and post-injection was determined and the percentage increase in SNR (%ΔSNR) from baseline (pre injection) calculated. After Gd-Hyd injection, the CDAHFD mice and the controls showed distinct differences in liver signal enhancement over the time course of the imaging. Liver signal was greatest at 5 min post injection and decreased at 15 and 25 min post Gd-Hyd injection. The area under curve (AUC) for the DCE-derived change in signal was also calculated and plotted as a function of time on diet (Fig. 5a) and demonstrated that signal enhancement remained reasonably constant in the control animals over the course of the diet but in the CDAHFD group the liver signal increased steadily up to 10 weeks before decreasing at week 14 (CDAHFD %ΔSNR AUC: 2 week = 2030 ± 530, 6 week = 2400 ± 710, 10 week = 3750 ± 360, 14 week = 2350 ± 210). Switching mice fed CDAHFD for 10 weeks to normal chow for 4 weeks significantly decreased the liver signal compared to mice that received CDAHFD for the entire 14 weeks (%ΔSNR AUC: 1530 ± 140, p = 0.012). To show that the liver enhancement was specific we also computed the change in liver-to-muscle signal ratio (ΔLMR), where LMR = SI_liver_/SI_muscle_ (SI = signal intensity), and ΔLMR = LMR_post _− LMR_pre_. The AUC characterized for the ΔLMR vs time on diet (Fig. 5b) shows that Gd-Hyd is specifically enhancing the livers of CDAHFD animals and that the trend in AUC for ΔLMR with disease progression is similar to the AUC for %ΔSNR data with a maximum liver-to-muscle signal enhancement at week 10. The image enhancement data are shown qualitatively in (Fig. 5c–j) where the pre-injection image is shown in greyscale and a difference image of the 25 min post-injection image—the pre-injection image is shown in color scale for representative animals for all time points and treatment groups. The largest signal change is apparent for the 10 week CDAHFD animal, Fig. 5h. The small punctate signal changes in the animals on normal diet (Fig. 5c,e,g) are likely a result of a slight misregistration of the subtraction images due to animal motion rather than areas of focal fibrogenesis.

**Figure 6:**
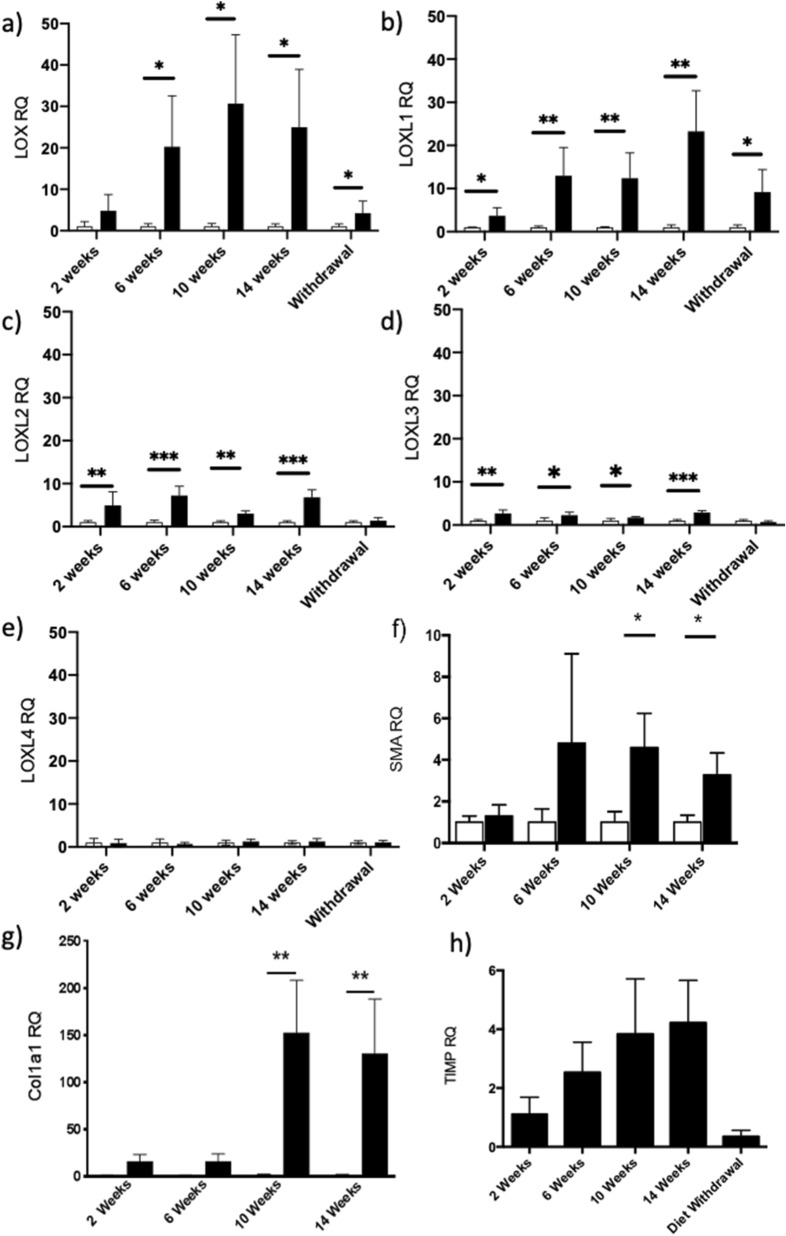
Lysyl oxidase gene expression in the CDAHFD model. Gene expression levels of (**a**) *Lox*, (**b**) *Loxl1*, (**c**) *Loxl2*, (**d**) *Loxl3*, and (**e**) *Loxl4* enzymes in normal chow (white bar) and CDAHFD groups (black bar) after 2, 6, 10, 14 weeks and switching mice fed CDAHFD to normal chow between week 10 and week 14. Expression levels are reported relative to normal chow data for each respective enzyme. Gene expression levels of (**f**) smooth muscle actin (*Acta2*) and (**g**) collagen, type 1, alpha 1 (*Col1a1*) in normal chow (white bar) and CDAHFD groups (black bar) at 2, 6, 10 and 14 weeks (relative to normal chow group). (**h**) Gene expression levels of fibrogenesis marker *Timp1* in CDAHFD groups at 2, 6, 10 and 14 weeks and after switching mice fed CDAHFD to normal chow between week 10 and week 14. (*P < 0.05, **P < 0.01, ***P < 0.001).

Lysyl oxidases are a family of enzymes that oxidize the terminal amino group of lysine to allysine, an essential step in collagen cross-linking. Since Gd-Hyd binds to allysine we also measured the expression of genes responsible for lysyl oxidase enzymatic activity: *Lox* and *Loxl1-4*. At all time points, *Lox* and *Loxl1-3* gene expression were elevated in mice fed the CDAHFD as compared to mice fed normal chow (Fig. 6). However, *Loxl4* expression remained largely unchanged in response to CDAHFD. *Lox* and *Loxl1* expression significantly increased over time on the CDAHFD, with *Lox* levels peaking at week 10 (31-fold increase compared to normal chow) and *Loxl1* at week 14 (23-fold increase compared to normal chow) (Fig. 6). Compared to mice fed CDAHFD for the entire 14 weeks, *Lox* and *Loxl1* expression decreased 5.8-fold and 2.5-fold, respectively, after switching mice fed CDAHFD for 10 weeks to normal chow for 4 weeks. The absolute expression level of *Lox* was higher than *Loxl1*-4 suggesting that Gd-Hyd imaging tracks more closely with *Lox* expression.

**Figure 7:**
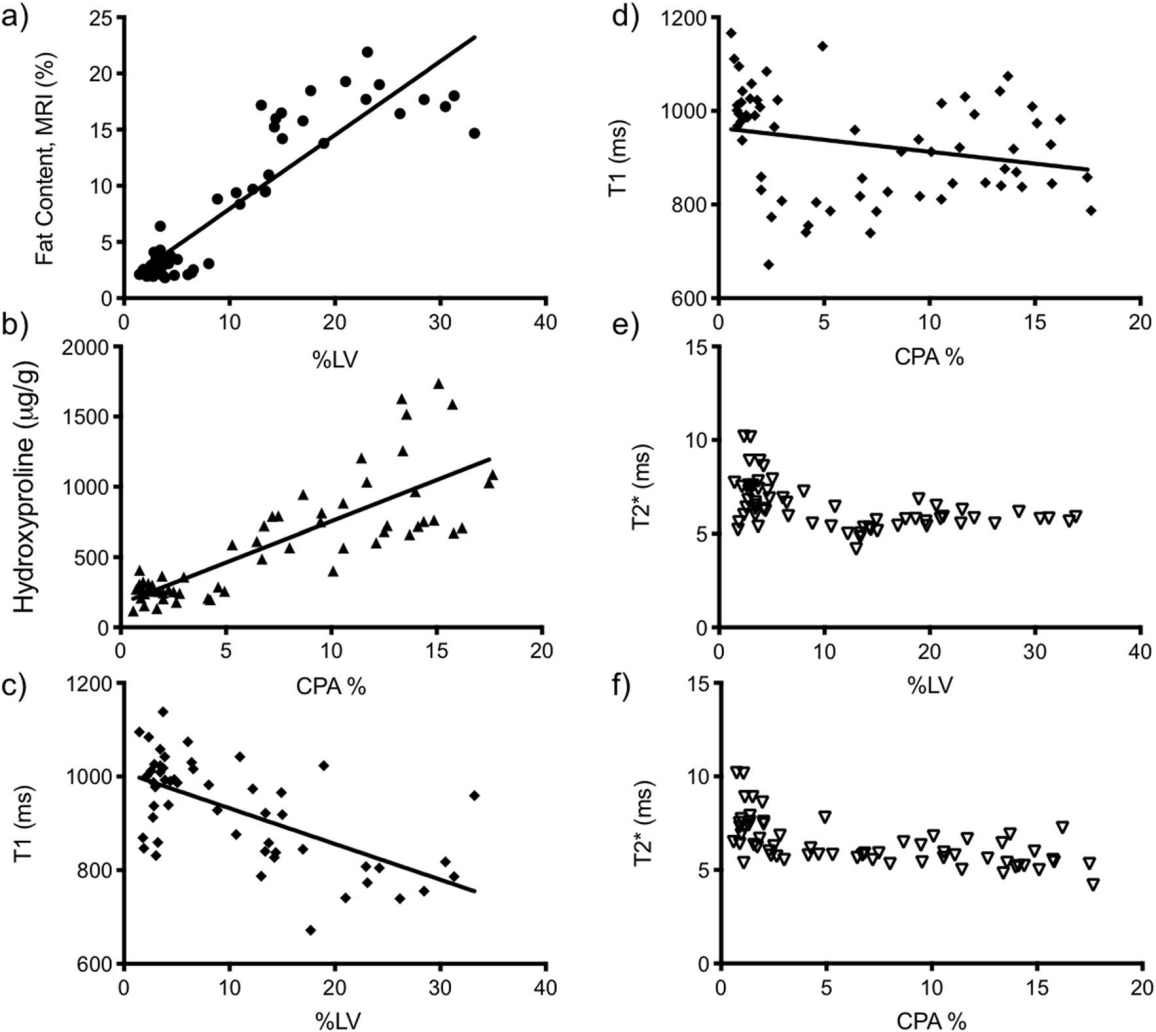
Correlation between ex vivo measures of disease and noninvasive imaging. Plots showing positive correlation between (**a**) MRI fat content (%) and LV% and (**b**) hydroxyproline concentration and CPA%; the negative correlation between (**c**) T1 and LV% and (**d**) T1 and CPA% and the lack of correlation between (**e**) T2* and LV% and (**f**) T2* and CPA%.

To determine whether existing non-invasive MRI measures like the Dixon method or T1 or T2* relaxation times could report on steatosis and fibrosis, we correlated these measurements with histological estimates of steatosis assessed by % lipid vacuolization (% LV) and fibrosis by the collagen proportional area (CPA), Fig. 7.

## Discussion

Biopsy, the current gold standard for NASH diagnosis, is an invasive procedure with inherent risks and not a practical solution for monitoring disease progression or response to therapy^50^. We reasoned that non-contrast MR imaging and molecular MR imaging of allysine could represent a direct and objective measure of NASH fibrosis.

While no animal model represents all aspects of human NASH, the CDAHFD model has become increasingly common for investigating novel therapeutics over the last few years given its ability to generate a robust fibrotic phenotype^51–54^. Here, we report for the first time a longitudinal, pathological characterization of the CDAHFD mouse model with respect to non-invasive imaging. We demonstrate that multiparametric MR assessment of the liver can reliably monitor changes in key pathological features like steatosis and fibrosis. Our results suggest that multiparametric MRI could not only be a useful tool for following disease progression in human patients in order to determine when to start treatment but also for monitoring the response to intervention.

Mice fed CDAHFD diet showed an immediate increase in steatosis with fat content build up within the vescicles of the hepatocytes leading to macrovesiculation and disruption of hepatocyte function. Over time, loss of hepatocytes in combination with increased myofibroblast activation and recruitment of immune cells leads to an overall decrease in total steatosis, increased inflammation and advanced fibrosis as observed in the human disease. Consistently, MR imaging (Dixon method) distinguishes this decrease in steatosis over time, with measurements of fat content in liver correlating with morphometric histologic methods (Fig. 7a, r = 0.90). Likewise, biochemical measurements (hydroxyproline analysis) of mice fed CDAHFD for 14 weeks distinguishes an increase in fibrosis over time, with total collagen burden correlating with morphometric methods (CPA analysis) (Fig. 7b, r = 0.82).

T1 times are shorter for mice fed CDAHFD compared to those that received normal chow, but increased over time. In fact, after 14 weeks there is little difference between the control and CDAHFD cohorts suggesting T1 shortening is sensitive to the fat content of tissue (Fig. 7c, r = − 0.64), and less indicative of the extent of fibrosis (Fig. 7d, r = − 0.26). This would be consistent with recent results in humans demonstrating that corrected T1 in multiparametric LiverMultiScan is unreliable for staging fibrosis^25^. T2* shows little dependence on the extent of either steatosis or fibrosis over time in this model, and is not indicative of the extent of disease (Fig. 7e,f).

The MRI probe Gd-Hyd which targets allysine was previously shown to be effective and specific for identifying liver fibrogenesis in a 12 week CCl_4_ disease model, with a highly significant increase in MR signal in the fibrotic liver tissue compared to control animals, that tracked with hydroxyproline concentration^36^. In this NASH model of advanced fibrosis, Gd-Hyd uptake is significantly increased in the livers of mice fed CDAHFD as compared to mice fed normal diet, but there is no direct correlation between Gd-Hyd liver signal enhancement and the extent of fibrosis. CPA and hydroxyproline levels are at a maximum at week 14 whereas the maximum Gd-Hyd signal enhancement is at week 10. Gd-Hyd signal enhancement did show a strong correlation with *Lox* expression, which also peaked at week 10 and decreased at 14 weeks. In the previous 12 week CCl_4_ study, Gd-Hyd signal enhancement similarly tracked with *Lox* gene expression with both Gd-Hyd signal and *Lox* expression maximally elevated at the 12 week timepoint^36^. A decrease in *Lox* expression would lead to a reduction in the amount of allysine formed, and the amount of Gd-Hyd bound to collagen. This might indicate that fibrogenesis has started to decrease leading to disease stabilization in the mouse CDAHFD model by 10 weeks but not the mouse CCl_4_ model by 12 weeks. Small decreases in *Acta2* expression (Fig. 6f), a marker of myofibroblast activation, and *Col1a1* expression (Fig. 6g) as well as stabilization of *Timp1* expression, a marker of fibrogenesis, between weeks 10 and 14 (Fig. 6h) further supports the decrease of active disease progression in the CDAHFD model. We observed a similar finding in a pulmonary fibrosis study where as the fibrosis (scar) matured, the allysine levels and LOX activity declined but hydroxyproline values remained elevated. In that study, Gd-Hyd imaging tracked with allysine/LOX activity as a readout of fibrogenesis^36^. Importantly, even though Gd-Hyd imaging decreased from weeks 10 to 14 as the disease stabilized, a further reduction was seen when animals fed CDHAFD for 10 weeks were switched to normal chow, suggesting that Gd-Hyd is a sensitive method for detecting reduced fibrogenesis during disease resolution. Importantly, additional decreases in *Acta2, Col1a1,* and *Timp1* expression were also noted on diet withdrawal.

The development of novel NASH treatments is restricted by the need for biopsy to monitor treatment response. There is therefore a need for noninvasive objective and quantitative biomarkers of treatment response. MRI would be an ideal modality for a number of reasons: it is non-invasive and provides whole liver coverage with the ability to detect and quantify disease heterogeneity; it does not involve ionizing radiation making it safe for repeated imaging which is important in a slow progressing disease where patients may be followed for decades; MRI readily scales from mouse to human such that MRI protocols can be developed preclinically and then translated to clinical trials. MRI is increasingly used in NASH drug development, and MRI measurements of liver fat and relaxation times are highly reproducible and repeatable^55–57^. MRE is very effective at detecting advanced fibrosis in patients^58,59^, however its ability to monitor treatment response in clinical trials is still to be established^58,59^. A multi-parametric MRI approach utilizing a fibroisis/fibrogenesis specific probe is therefore most likely to give an accurate treatment assessment.

This study builds upon prior work with Gd-Hyd enhanced MRI. Chen et al. showed in the bleomycin injured mouse model of pulmonary fibrosis that Gd-Hyd imaging of allysine reflected fibrogenesis and that Gd-Hyd could distinguish active fibrogenesis from stable scar, and also showed that Gd-Hyd could monitor treatment response with the pan lysyl oxidase inhibitor beta-aminopropionitrile^36^. They also showed that Gd-Hyd could be used to image liver fibrogenesis in the mouse CCl_4_ model as well as the resolution in liver fibrosis when CCl_4_ was withdrawn. Zhou et al. used Gd-Hyd to measure hepatic fibrogenesis in the rat CDAHFD model and showed that Gd-Hyd enhanced MRI was superior to collagen-targeted molecular MRI, to magnetic resonance elastography, and to native T1 in measuring response to treatment with either elafibrinor or diet change^26^. Ferreira et al. recently showed using different transgenic mouse models of parasitic infection that Gd-Hyd enhanced MRI was sensitive to fibrogenesis but not to the presence of inflammation^60^.

Gd-Hyd enhanced MRI is expected to be a good noninvasive measure of the rate of fibrosis, however fibrogenesis may not necessarily correlate with disease stage, as was shown here. Especially at very advanced stages of disease, the rate of fibrogenesis may not be reflective of overall fibrotic burden and other methods such as ultrasound^61^ or MR elastography^62^ or emerging molecularly targeted approaches^63,64^ may be considered for noninvasive staging of disease. In very advanced disease, altered liver perfusion may also need to be considered for quantitative molecular imaging.

Baseline non-contrast fat MR imaging followed by contrast Gd-Hyd enhanced MRI represent a promising multi-parametric approach for the non-invasive detection and staging of steatosis and fibrogenesis, and for monitoring treatment response for patients with NASH. Gd-Hyd is a derivative of the stable, clinical contrast agent gadoterate and is well suited for clinical translation. Target localization with Gd-Hyd is fast after injection and the contrast agent was previously shown to be rapidly and completely eliminated into the urine. Moreover, the imaging performed here utilized standard T1-weighted protocols that are available on commercial clinical scanners. While additional pre-clinical safety studies are required before commencing human studies, the results presented here indicate that these translational studies are warranted.
