# Author Correction: Quantitative, noninvasive MRI characterization of disease progression in a mouse model of non-alcoholic steatohepatitis

**DOI:** 10.1038/s41598-021-96648-2

**Published:** 2021-09-07

**Authors:** Philip A. Waghorn, Diego S. Ferreira, Derek J. Erstad, Nicholas J. Rotile, Ricard Masia, Chloe M. Jones, Chuantao Tu, Mozhdeh Sojoodi, Yin-ching I. Chen, Franklin Schlerman, Jeremy Wellen, Robert V. P. Martinez, Kenneth K. Tanabe, Bryan C. Fuchs, Peter Caravan

**Affiliations:** 1 Department of Radiology, Massachusetts General Hospital, Athinoula A. Martinos Center for Biomedical Imaging, Institute for Innovation in Imaging, Harvard Medical School, 149 13th St., Boston, MA 02129 USA; 2 Division of Surgical Oncology, Massachusetts General Hospital Cancer Center, Harvard Medical School, Boston, MA 02114 USA; 3 Pfizer, Cambridge, MA 02139 USA; 4 Present Address: School of Pharmacy, Universidade Federal de Minas Gerais, Av. Presidente Antônio Carlos, 6627, Pampulha, Belo Horizonte, Minas Gerais Brazil

Correction to: *Scientific Reports* https://doi.org/10.1038/s41598-021-85679-4, published online 17 March 2021

The original version of this Article contained an error in Figure 1, where the chemical structure mistakenly featured a pendant amine instead of a pendant hydrazide group. The original Figure 1 and accompanying legend appear below.

**Figure 1:**
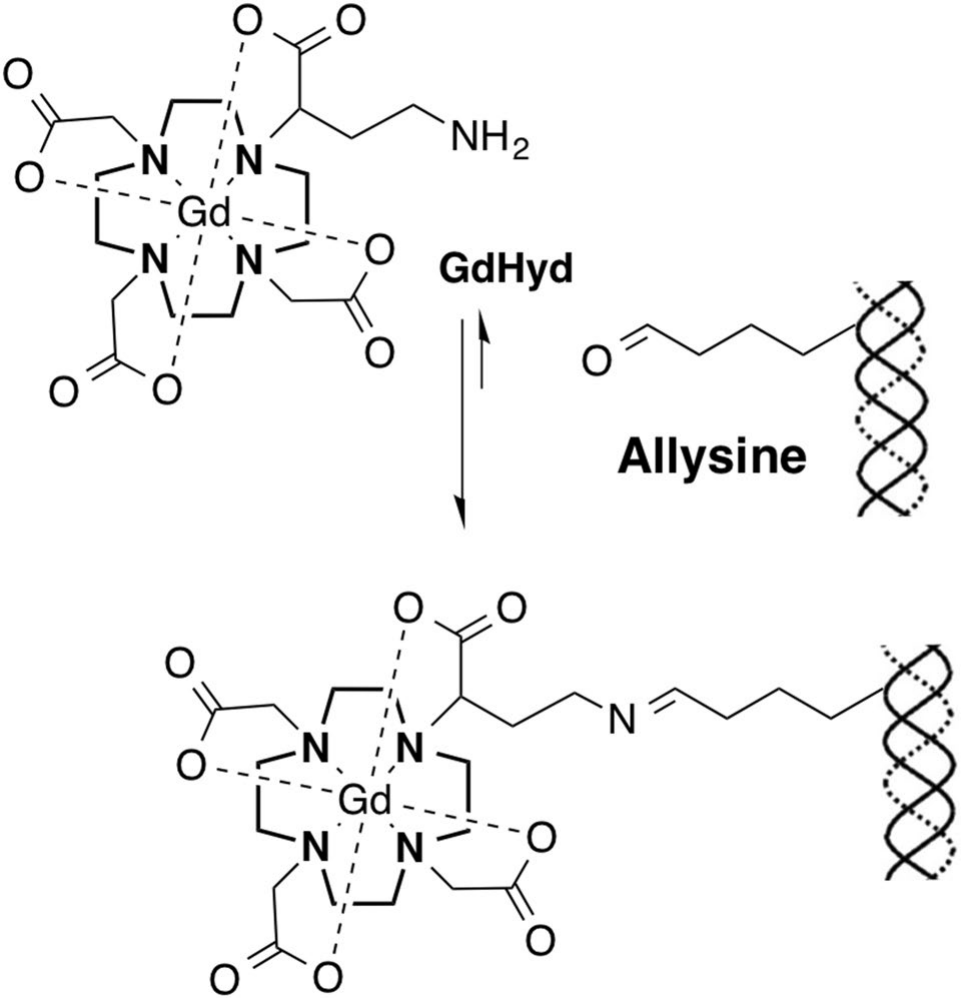
Structure of Gd-Hyd. Gd-Hyd is a water soluble, low molecular weight, extracellular gadolinium-based imaging agent functionalized with a hydrazide moiety for binding allysine on collagen.

The original Article has been corrected.

